# Macroscopical, Microscopical and Histochemical Analysis of *Eryngium karatavicum* Iljin Growing on the Territory of South Kazakhstan

**DOI:** 10.3390/plants12142714

**Published:** 2023-07-21

**Authors:** Meruyert Amantayeva, Kaldanay Kozhanova, Gulnara Kadyrbayeva, Aigul Medeshova, Yerbolat Tulebayev, Moldir Zhandabayeva, Gulnur Yeleken, Zoya Allambergenova, Szilvia Czigle

**Affiliations:** 1 School of Pharmacy, Kazakh National Medical University, Tole bi 94, 050012 Almaty, Kazakhstan; amantaeva.meruert@kaznmu.kz (M.A.); kozhanova.k@kaznmu.kz (K.K.); kadyrbaeva.g@kaznmu.kz (G.K.); zhandabaeva.m@kaznmu.kz (M.Z.); eleken.g@kaznmu.kz (G.Y.); allambergenova.z@kaznmu.kz (Z.A.); 2 School of Pharmacy, Karaganda Medical University, Gogol 40d, Karaganda 100000, Kazakhstan; medeshova@qmu.kz (A.M.); tulebaev@kgmu.kz (Y.T.); 3 Department of Pharmacognosy and Botany, Faculty of Pharmacy, Comenius University Bratislava, Odbojárov 10, SK-832 32 Bratislava, Slovakia

**Keywords:** *Eryngium karatavicum* Iljin, macroscopical analysis, microscopical analysis, histochemical analysis

## Abstract

Carrying out macroscopical and microscopical analyses of plants allows determining the species and identifying diagnostic signs of the plant that distinguish the studied object from other related species. Endemic plant species are a specific component of the flora, whose representatives grow in a relatively limited area, represented by a small geographical area. Their diagnostic morphological and anatomical data are insufficiently studied. Such endemic unexplored plant species include *Eryngium karatavicum* Iljin, which grows in the territory of South Kazakhstan. This article presents the results of macroscopical, microscopical and histochemical analyses of leaves, flowers and stems of *Eryngium karatavicum*. The results of morphological analysis of *Eryngium karatavicum* showed that the plant has distinctive features of macroscopical, microscopical and histochemical signs on the upper and lower sides of the leaf, stem, inflorescence, leaves of the wrapper and flower. These results can be used to confirm the authenticity, identification, and standardization of aerial parts of the endemic plant *Eryngium karatavicum* Iljin.

## 1. Introduction

*Eryngium* L. [1] is a genus of perennial plants (rarely biennial and summer) of the Umbrella family (Apiaceae, Saniculoideae) [2,3,4]. Genus *Eryngium* L. have about 250 species native to Eurasia, North Africa, North and South America, and Australia [5,6]. It is the largest and possibly the most taxonomically complex genus of the Apiaceae family [2]. Preparations of different *Eryngium* species are used in folk medicine, as they have the following therapeutic actions: antioxidant [7,8,9,10], anti-inflammatory [11], cytotoxic [10,12,13,14,15], antibacterial [16,17,18,19,20,21], antifungal [19], diuretic [11], antiallergic [11], etc. Today, the need to develop the technology of medicines from raw materials of unexplored endemic medicinal plant species of Kazakhstan is an urgent task of domestic pharmacy [22].

Biologically active compounds of Eryngii herba and Eryngii radix (*E. campestre* L.) are saponins, chlorogenic and rosmarinic acid, dihydropyranocoumarine derivatives [23]. Eryngii maritinii radix (*E. maritimum* L.) contains mucilage and saponins [23]. Eryngii plani herba and Eryngii plani radix (*E. planum* L.) contain flavonoids and triterpene saponins [23].

**Figure 1:**
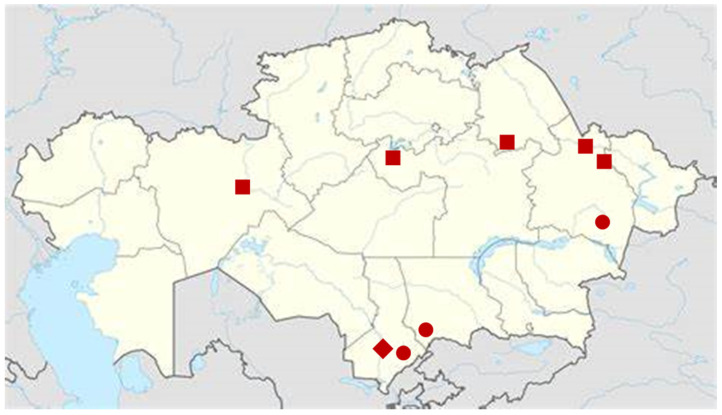
Geographical distribution of *Eryngium macrocalyx* Schrenk (●), *Eryngium planum* L. (■) and *Eryngium karatavicum* Iljin (◆) in Kazakhstan, based on [25].

It is also known that many promising plant species for medicine have been little studied from the standpoint of botanical resource studies and pharmacognostic analysis. Such plants also include species of the genus *Eryngium* L. [24]. In our country, there are some species of genus *Eryngium*, such as *Eryngium caucasicum* Trautv., *Eryngium macrocalyx* Schrenk, and *Eryngium planum* L. [25]. At the same time, it should be noted that Southeastern Kazakhstan is the leader in the distribution of endemic plant species, 270 out of 776 endemes. The second place is occupied by South Kazakhstan [26]. The distribution of *Eryngium* L. is presented below (Figure 1).

Foreign and domestic scientists have shown interest in studying the morphology and anatomy of some species of the genus *Eryngium* L. Pharmacognostic signs of plant raw materials of *Eryngium karatavicum* have not been studied and, therefore, there is no regulatory documentation. For the use of new types of plant raw materials in pharmacy, including our facility, their standardization is necessary. Macroscopic and microscopic analyses are necessary to identify medicinal plants [22,27,28,29,30,31]. To date, there are many research methods that allow us to assess the belonging of medicinal plants to a particular group. Such methods include histochemical analysis [32,33,34], which allows us to identify the presence of biologically active substances and their localization in tissues and organs. Medicinal plants contain many secondary metabolites, such as phenolic substances, tannins, flavonoids, alkaloids, polysaccharides, etc. These secondary metabolites are in great demand in the pharmaceutical industry and have a different range of pharmacological activity.

In this regard, the study of macroscopical, microscopical, and histochemical signs is necessary for the identification of raw materials and for further research in the field of pharmacy and medicine.

The purpose of our work is to determine the macroscopical, microscopical and histochemical characteristics of the raw materials of *Eryngium karatavicum* plants growing on the territory of the Karatau mountain range in South Kazakhstan for their further identification.

## 2. Results and Discussion

**Figure 2:**
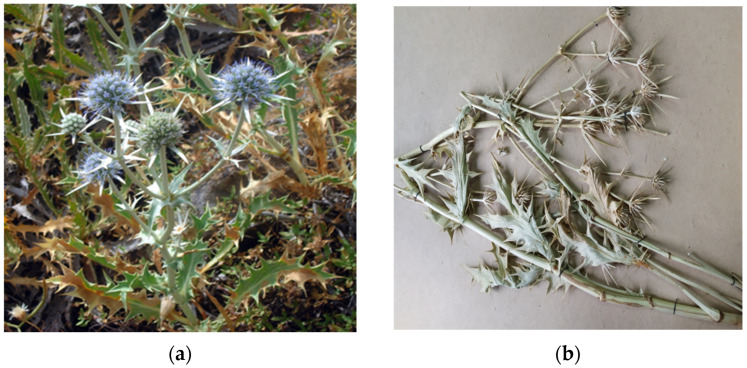
*Eryngium karatavicum* Iljin growing in nature (**a**) and dried whole aerial parts—raw material (**b**).

The objects of this study were the aerial parts of *Eryngium karatavicum* Iljin, collected in the territory of the Syrdarya-Turkestan State Regional Natural Park (South Kazakhstan) during the flowering period in June 2020 (Figure 2).

The results of the macroscopical analysis of raw materials of *Eryngium karatavicum* Iljin are presented.

Description of the appearance: *Eryngium karatavicum* Iljin (Figure 2) is a perennial herbaceous plant up to 30 cm high. The root is cylindrical, the root neck is lignified, branched into particles, and covered with fibrous remnants of last year’s leaves. The stems are few, rounded, furrowed, and slightly branched in the upper part (Table 1). The leaves are leathery, smooth on top, with convex reticulated venation on the bottom. The lower leaves are collected in a dense basal rosette, sitting on short petioles. The plate is oblong-lanceolate, narrowed to the base into a petiole; the plate itself is pinnately incised into triangular and lanceolate prickly lobes. The stem leaves are bent downward, pinnately divided almost to the middle of the vein into lanceolate prickly lobes, and at the base expanded into prickly sheaths. The flowers are collected in head-shaped inflorescences, 1.2–2 cm long, wrapping leaves 6–7 (11), they are almost flat, smooth, and straight, linear-awl-shaped, and prickly.

The results of the morphological analysis of *Eryngium karatavicum* Iljin, presented in Table 1, show that the plants have distinctive macroscopic characteristics of the structure.

*Eryngium* L. species grow in many countries around the world. Table 2 shows the comparative characteristics of three species of this plant (*E. planum* L., *E. babadaghense* G. E. Genç, Akaln & Wörz, *E. karatavicum* Iljin).

The detected macroscopic characteristics (Table 1 and Table 2) were compared with the data in the literature [37,38,39,40].

Results of the microscopical analysis of raw materials of *Eryngium karatavicum* Iljin

### 2.1. Leaf Surface

**Figure 3:**
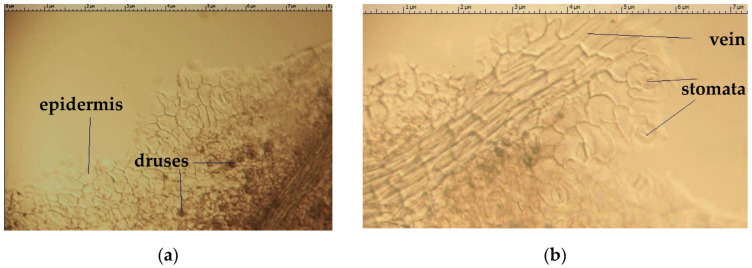
Upper (**a**) and lower (**b**) epidermis of *Eryngium karatavicum* Iljin (surface preparation).

The sheet of *Eryngium karatavicum* Iljin is flat, surrounded on both sides by a single-layer epidermis. Its cells are round or oval in shape, with slightly sinuous walls, thickened on the outside; covered with a layer of cuticle, with folds forming around the stomata (Figure 3).

Above the leaf veins, the epidermis consists of rectangular cells tightly adjacent to each other. Stomata are numerous, located on both sides of the leaf (amphistomatic leaf type), diacytic type (one stomata is surrounded by two cells of the main epidermis), there are 8 to 16 stomata per 1 mm^2^. The stomatal index (lower surface) by [41] is 15.0–18.0 per 1 mm^2^. The stomata themselves are broadly oval in shape and consist of bean-shaped periosteal cells. The pubescence on the leaf surface was not detected, and calcium oxalate druses were observed (Figure 3).

The stomatal structures of Apiaceae leaves from 20 genera and 29 species (9 genera and 11 species of subfamily Saniculoideae, 11 genera and 18 species of basal subfamily Apioideae) are varied; three types of stomata are observed, anomocytic, anisocytic, and paracytic [41]. The stomata structures of Saniculoideae are similar to those of Apioideae. The stomata of Saniculoideae are anomocytic (35–75%) and anisocytic (25–65%) [41]. The genus *Eryngium* has all three types of stomata. The stomata of subfamily Apioideae are more anomocytic (75–100%) and less anisocytic (5–25%) [41]. Morphological stomatal types in leaves of 119 species belonging to 3 subfamilies of the Apiaceae family have been studies [42]. Anomocytic, hemiparacytic, brachiaparacytic, diacytic (including diallelocytic), paracytic (including parallelocytic) types were found. In many cases, 2–3 stomata types are found on one leaf. In our samples, the diacytic type of stomata was typical.

### 2.2. Transverse/Cross Section

**Figure 4:**
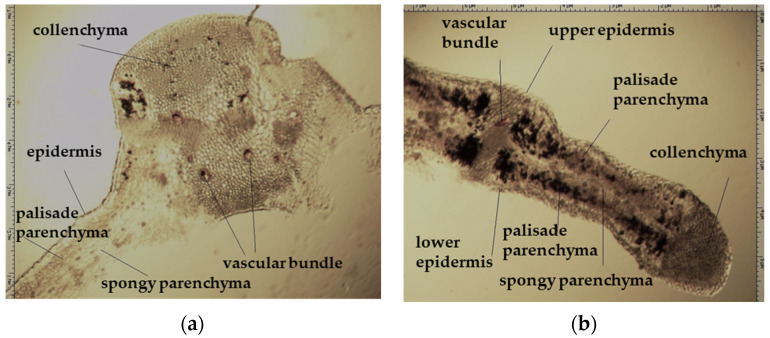
Transverse section of *Eryngium karatavicum* Iljin leaf: (**a**) fragment through the main vein; and (**b**) lateral fragment.

On the transverse section, the leaf of *Eryngium karatavicum* Iljin is flattened, of the isolateral type (Figure 4), with protruding areas of the leaf veins on the lower side.

The cells of the upper and lower epidermis in the cross-section are rounded-rectangular and dense. The areas of living mechanical tissue of the collenchyma were marked around the leaf veins and at the ends. The main and lateral veins consist of a xylem thread oriented to the underside and a phloem thread. The handles are reinforced with collenchyma on both sides. The vascular bundle is a collateral, closed type. The mesophyll differentiates into columnar and spongy tissues. The mesophyll of the stockade is located between of 2 layers, both from the lower and upper sides. The central part between the layers of palisade parenchyma is filled with spongy parenchyma. In the pulp of the leaf, single calcium oxalate druses were observed, as well as red vascular bundles (Figure 4).

**Figure 5:**
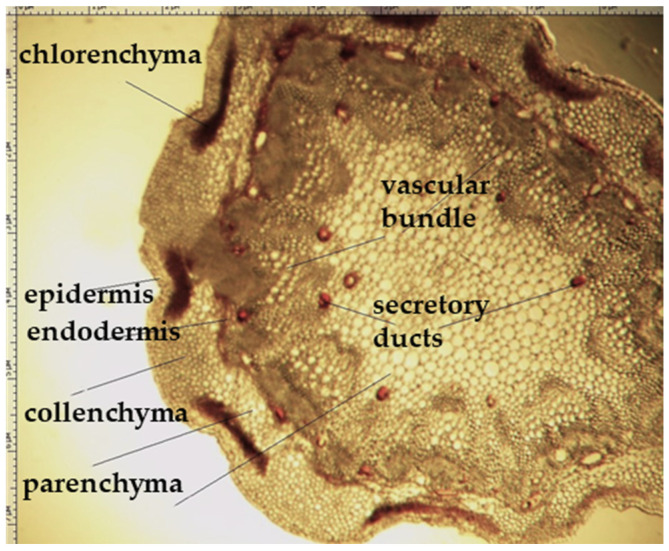
Cross section of the stem of the *Eryngium karatavicum* Iljin.

The stem of *Eryngium karatavicum* Iljin is rounded-lobed in a cross section (Figure 5). A single layer epidermis consisting of rounded cells (almost in the form of beads) is located along the perimeter. At the corners of the stem, corresponding to the lobes, there are significant areas of angular collenchyma, dark-colored oval or elongated zones of chlorenchyma are located between them. The next zone is represented by a multilayer cortical parenchyma, vascular bundles (phloem + xylem) forming a ring (typically dicotyledonous).

The conducting zone is separated from the primary cork by a single layer endoderm. Its cells are thin-walled, oval, and more elongated in shape. The conducting zone of the beam type. The vascular bundles are collateral, closed, oval to triangular cone-shaped. The sizes of the vascular bundles differ from each other. The bundles consist of phloem (closer to the periphery) and xylem, reinforced with small sections of the sclerenchyma. The central part is filled with loose cells of the core parenchyma. In the conducting zone, there are numerous secretory ducts with essential oil (schizogenic type), painted red-brown, rounded or oval shape.

### 2.3. Histochemical Signs

As a result of the study, a characteristic staining of cells of different types was revealed, which is characterized by the result of the interaction of reagents with detectable metabolites [32,33,34]. The results of histochemical analysis to identify certain groups of metabolites in the aboveground organs of the studied plant are presented in Table 3.

**Figure 6:**
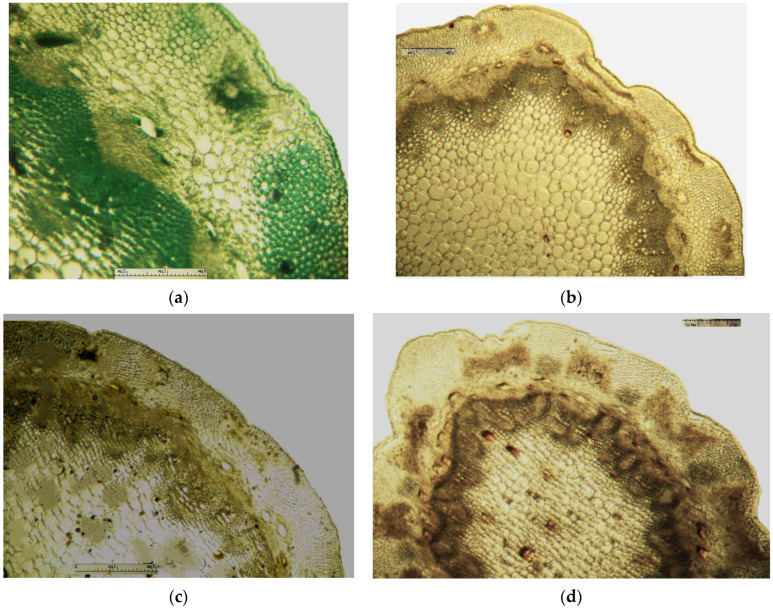
Results of histochemical study of the stem of the *Eryngium karatavicum* Iljin. (**a**) Stem, cross section, fragment; color—methylene blue reagent. (**b**) Stem, cross section, fragment; color—ferric chloride reagent. (**c**) Stem, cross section, fragment; color—potassium bichromate reagent. (**d**) Stem, cross section, fragment; color—thymol (in concentrated H_2_SO_4_).

The results of the histochemical examination of the stem (Figure 6) showed that staining is observed of individual tissue groups, indicating the presence of essential oil, phenolic compounds (tannins, flavonoids), and polysaccharides.

The presence of essential oil in the epidermis of the stem (Figure 6), chlorenchyma, in the receptacles and individual drops in secretory cells was observed.

Phenolic compounds have been identified at the following sites: stem chlorenchyma, vascular bundles, sclerenchyma, separate sections of the cortex parenchyma (Figure 6).

The presence of flavonoids was observed in all stem cells (Figure 6), but the greatest accumulation was observed in the sclerenchyma, a smaller content—in cells of the angular collenchyma, chlorenchyma and cortex parenchyma.

Polysaccharides are marked locally in the cells of the endoderm and cortex parenchyma in stem (Figure 6).

Typical staining for alkaloids and starch has not been recorded in the stem (Figure 6).

**Figure 7:**
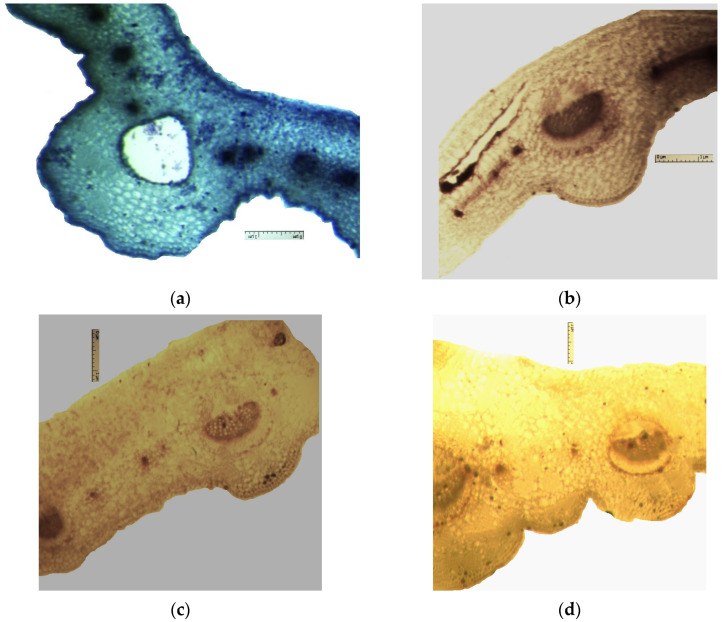
The results of the histochemical study of the leaf of the *Eryngium karatavicum* Iljin. (**a**) Leaf, cross section, fragment; color—methylene blue reagent. (**b**) Leaf, cross section, fragment; color—ferric chloride reagent. (**c**) Leaf, cross section, fragment; color—potassium bichromate reagent. (**d**) Leaf, cross section, fragment; color—thymol (in concentrated H_2_SO_4_).

Similar studies were conducted for the leaf (Figure 7) of blue-headed *E. karatavicum*. Also, as for the stem, the accumulation of phenolic compounds (tannins, flavonoids), essential oil, and polysaccharides was observed, and starch and alkaloids were not detected.

Essential oil (Figure 7) is localized in cells of the secretory ducts, to a lesser extent, in individual secretory cells of the mesophyll. Phenolic compounds (Figure 7) are observed in cells of the epidermis, collenchyma, vascular bundles. The localization of flavonoids (Figure 7) was observed in mesophyll cells. Polysaccharides (Figure 7) are fixed in individual mesophyll cells of the leaf, too.

## 3. Materials and Methods

### 3.1. Materials

The objects of this study were the aerial parts of *Eryngium karatavicum* Iljin, collected on the territory of the Syrdarya-Turkestan State Regional Natural Park (South Kazakhstan) during the flowering period in June 2020 (Figure 2). The species was identified by the Director of the Institute of Botany and Phytointroduction, Sitpayeva Gulnara, on 25 July 2019, and a certificate of identification confirmation was issued (01-08/200). Herbarium samples were deposited at the Institute of Botany and Phytointroduction (Kazakh National Medical University, Kazakhstan).

### 3.2. Macroscopical (Morphological) Analysis

Samples of *Eryngium karatavicum* Iljin were photographed using a Levenhuk DTX 50 microscope (Levenhuk Optics, Praha, CZ), monocular, planachromatic objective lens, zoom objective 20×, tube ½; digital camera: 1.3 Mpix; software: Microcapture Basic vers. 3.1.1. Additional drawing program: Paint vers. 10.0. When studying the morphological parameters of plants, the shape of the stem, leaf, flower, and the degree of pubescence, the color of individual elements were considered.

### 3.3. Microscopical Analysis

Samples of plant raw materials (leaves and stems) of *Eryngium karatavicum* Iljin were fixed in a mixture of glycerin: ethanol 96% (*v*/*v*): distilled water in a ratio of 1:1:1 (Ostrich and Fleming mixture) [32,33]. Cross sections of the leaf, petiole, and stem were made by cryostat MEV (SLEE Medical, Mainz, DE) with 24 freezing positions, a cryochamber cooling temperature down to −35 °C, and by rotary microtome ROTMIK-1 (OrionMedic, St. Petersburg, RU). For the stem, micropreparations were made from the middle part of generative shoots; for the leaf, the central part of the fragment of the final leaflet; for the petiole, the middle. Slices were purified with glycerin. The preparations were photographed with an optical microscope: LEICA (Leica, Wetzlar, D) DME, trinocular, planachromatic objective lens, zoom objective 20×, tube ½; digital camera: LEICA EC 3 Mpix; software: LEICA application suite 2.4.0 R1, LAS EZ ver. 1.3.0 and with the Altami BIO 8 microscope (Altami, St. Petersburg, RU) with a 3.1 megapixel digital camera, magnification 16 × 4, 16 × 10 and 16 × 45. Photo-processing and measurements of micro-preparations were carried out in the Altami Studio program, vers. 4.0 RC, later using Paint 10.0. When describing the anatomical structure, the principles set out in the works of [27,29] are applied.

### 3.4. Histochemical Analysis

The dry raw materials were soaked and preserved in a mixture of ethanol 70% (*v*/*v*)/glycerin/distilled water in a ratio of 1:1:1 (Strauss-Fleming solution) [32,33].

Histochemical analysis was performed for cross-sections of the stem and leaf. During histochemical analysis, we used the following reagents [32,33,34]:

- Methylene blue reagent (Sigma-Aldrich, St. Louis, MO, USA) for identification of phenol (essential oil compounds);
- 10% thymol reagent (Sigma-Aldrich, St. Louis, MO, USA) solution in concentrated H_2_SO_4_ (Centralchem, Bratislava, Slovakia) for identification of polysaccharides;
- Lugol’s reagent, Iodine-Potassium Iodide reagent (Centralchem, Bratislava, SK) for the identification of starch;
- 10% K_2_Cr_2_O_7_ reagent (ethanolic) (Centralchem, Bratislava, SK) for the identification of phenolic compounds—tannins;
- 3% FeCl_3_ reagent (ethanolic) (Centralchem, Bratislava, SK) for identification of phenolic compounds—flavonoids;
- Dragendorff’s reagent, K(BiI_4_) (Sigma-Aldrich, St. Louis, MO, USA) for identification of alkaloids.

A change in the color of certain tissues served as a sign of the localization of certain groups of metabolites in the tissues of *Eryngium karatavicum* Iljin. The photos were edited in the Paint 10.1 and Krita 5.0.6 programs (the price of dividing a microline is 10 microns).

## 4. Conclusions

For the first time, diagnostic signs of the aerial parts of the *Eryngium karatavicum* Iljin plant were determined at the macroscopic and microscopic levels.

For the first time, the following diagnostic signs were identified at the macroscopic level:

- The upper side of the leaf surface is rough, hard, with pronounced veins; the surface is without pubescence, the edges are thickened, the tips of the lobes are pointed, the color is green, and in the area of the veins they are white-yellowish, while the dots are yellow-reddish;
- The lower side of the leaf surface is hard and rough; the veins protrude above the surface, the color is green, in the area of the veins it is white and yellowish, prickly—yellow-reddish;
- The stem is rounded or oval in cross-section, the surface of thin stems is smooth, thicker ones are ribbed, round veins protrude above the surface, the surface is smooth, the color is light green, at the break with red or orange stripes corresponding to the ribs;
- The inflorescence is glabrous, with 6–11 leaflets;
- Leaflet (wrapped sheet) is awl-shaped, triangular, with a well-defined middle vein, the veins protrude above the surface; the surface is smooth, without pubescence, the tip is pointed, the surface is light green, and at the tip of the wrapped leaflet it has a reddish tint;
- A flower of conical shape, with five leaves fused at the base, the tips are reduced to a short, pointed point; a median vein protruding to the surface is marked on the surface; the flower color is light green, almost white in the area of the middle vein.

For the first time, the following diagnostic signs were detected at the microscopic level:

- The shape of the cells of the main epidermis of the leaf, and the presence of stomata of the diacytic type;
- An isolated type of leaf with a multilayered epidermis on the upper and lower sides of the leaf;
- The presence of calcium oxalate druse, translucent from the surface of the leaf;
- The presence of small secretory ducts with essential oil on the cross-section of the leaf and stem.

We identified in *E. karatavicum* by histochemical analysis (in stem and leaves) phenolic compounds (tannins, flavonoids), essential oil, and polysaccharides, but starch and alkaloids were not detected.

The macroscopic, microscopic and histochemical analysis of the *E. karatavicum* Iljin aerial part is useful in standardization for sample identification.

## Figures and Tables

**Table 1:** Morphological parameters of aerial parts of *Eryngium karatavicum* Iljin.

Plant Organ		Description
The upper side of the leaf	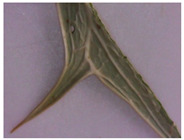	The leaf on the upper side is rough and hard, with pronounced veins; the surface is without pubescence. The edges are thickened, and the tips of the lobes are pointed. The color is green, in the area of the veins it is white-yellowish, and the dots are yellow-reddish.
The underside of the leaf	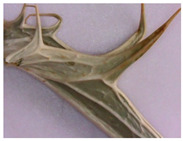 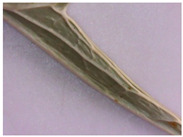 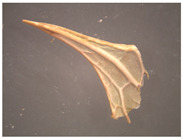	On the underside, the surface is hard and rough; the veins protrude above the surface. The color is green, in the area of the veins it is white-yellowish, and the dots are yellow-reddish.
Stem	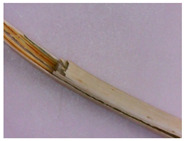 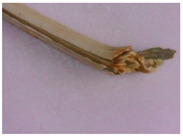 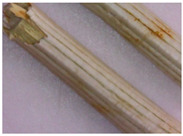	The stems are rounded or oval in cross-section, the surface of the thin stems is smooth, and the thicker ones are ribbed. The veins are rounded and protruding above the surface. The surface is smooth. The color is light green, broken, with red or orange stripes corresponding to the edges.
Inflorescence	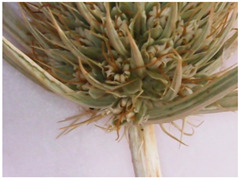 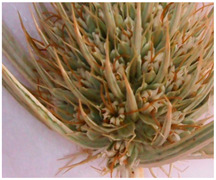	Inflorescence—glabrous, with 6–11 leaflets (wrapped leaf).
Wrapper leaf	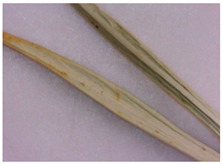 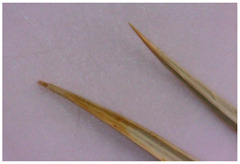	The leaflets are awl-shaped, triangular, with a well-defined middle vein. Veins protrude above the surface. The surface is smooth, without pubescence. The end is pointed. The surface is light green, with a reddish tinge at the tip of the wrapping leaflet.
Flower	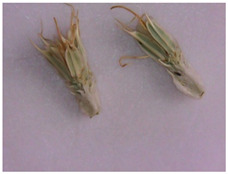 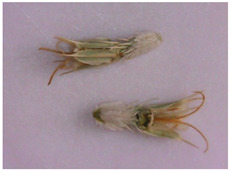	The flower is conical in shape, with 5 fused leaves at the base, the tips of which are collected in a short peak. On the surface, there is a median vein protruding from the surface. The color is light green, almost white in the area of the middle vein.

**Table 2:** Comparative morphological features of some species of *Eryngium* L. (Apiaceae).

*Eryngium* sp. Specifications	*E. planum* [35]	*E. babadaghense* [36]	*E. karatavicum*
Raw material form	Aerial part.	Aerial part.	Aerial part.
Stem morphology	Complex, rigid, spiral leaf arrangement.	Oval in cross section.	Rounded or oval in cross section, the surface of the thin stems is smooth, the thicker ones are ribbed, the veins are round—protrude above the surface, the surface is smooth.
The size of the stem and individual branches	Generative shoots, length 10–100 cm, vegetative shoots up to 30 cm, diameter 0.2–12 mm.	Generative stems 75 cm high, narrow wedge shaped at the base, 9–24 × 10–14 cm.	Generative shoots up to 100 cm, vegetative shoots up to 20–25 cm, diameter up to 7–8 mm.
The nature of the surface, the color of the stem and branches	The stems are glabrous, rounded, smooth, or slightly striated. In the lower part, the stems are grayish-green, and in the upper part they are purplish-bluish.	The underside is bluish, numerous, and the plates are obovate.	The stems are rounded or oval in cross-section, the surface of the thin stems is smooth, and the thicker ones are ribbed. The veins are rounded and protruding above the surface. The surface is smooth. The color is light green, broken, with red or orange stripes corresponding to the edges.
Macroscopic signs of leaves	The basal leaves are simple whole, oblong, oval, elliptical, or oblong–ovate, obovate, with a leaf blade length of up to 15 cm, width 2–7 cm.	Three to four palmately dissected linear segments, 2–3 mm wide, terminal segments longer than the lateral ones.	The leaf on the upper side is rough and hard, with pronounced veins; the surface is without pubescence. The edges are thickened, and the tips of the lobes are pointed. Color—green, in the vein area—white-yellow, dots—yellow-reddish.
Type of inflorescence and flowers, their morphological structure and signs	Corymbose, thyrsus, private—the head is ovoid about 15 mm long. Bracts 5–6 mm long, thinly pointed, oblong-triangular. The flowers are small, actinomorphic, have a five-membered calyx, the teeth of the calyx are oblong-pointed elongated into a long (about 2 mm) spike, leaf-shaped, green, protruding.	Two-phase or corymbose inflorescence.	Inflorescence—glabrous, with 6–11 leaflets (wrapped leaf). The flower is conical in shape, with 5 fused leaves at the base, the tips of which are collected in a short peak. On the surface there is a median vein protruding from the surface. The color is light green, almost white in the area of the middle vein.

**Table 3:** Comparative histochemical features of some species of *Eryngium* L.

Defined Component	Reagent	Coloring	Stem	Leaf
Essential oil	methylene blue reagent	blue-violet	+	+
Polysaccharides	10% thymol reagent	orange-red	+	+
Starch	Lugol’s reagent	blue, purple	−	−
Phenolic (tannins) compounds	10% K_2_Cr_2_O_7_ reagent	brown, yellow	+	+
Phenolic (flavonoids) compounds	3% FeCl_3_ reagent	black, blue, green	+	+
Alkaloids	Dragendorff’s reagent	black, brown-green	−	−

Note: + positive reaction; − negative reaction.

## Data Availability

All tables and figures are created by the authors. All sources of information are adequately referenced. It is not necessary to obtain copyright permissions.
